# Case report: Malignant transformation of ovarian endometrioma during long term use of dienogest in a young lady

**DOI:** 10.3389/fonc.2024.1338472

**Published:** 2024-01-31

**Authors:** Yi-Ting Chang, Ting-Fang Lu, Lou Sun, Yu-Hsiang Shih, Shih-Tien Hsu, Chin-Ku Liu, Sheau-Feng Hwang, Chien-Hsing Lu

**Affiliations:** ^1^ Department of Gynecology and Obstetrics, Taichung Veterans General Hospital, Taichung, Taiwan; ^2^ Department of Food and Nutrition, Providence University, Taichung, Taiwan; ^3^ College of Health Care and Management, Chung Shan Medical University, Taichung, Taiwan; ^4^ Center for General Education, Ling Tung University, Taichung, Taiwan; ^5^ School of Medicine, China Medical University, Taichung, Taiwan; ^6^ Department of Animal Science and Biotechnology, Tunghai University, Taichung, Taiwan; ^7^ Department of Palliative Care Unit, Taichung Veterans General Hospital, Taichung, Taiwan; ^8^ Institute of Biomedical Sciences, Ph.D. Program in Translational Medicine, and Rong Hsing Research Center for Translational Medicine, National Chung Hsing University, Taichung, Taiwan

**Keywords:** dienogest, endometrioma, endometriosis, ovarian cancer, clear cell carcinoma

## Abstract

Endometriosis is a benign disease, which is also regarded as a precursor to ovarian malignancy. Dienogest is a progestin treatment for endometriosis with efficacy and tolerability. A 35-year-old Taiwanese lady with ovarian endometrioma had taken dienogest for the last 5 years. During sonographic follow-up, surgery was suggested owing to suspicious of malignant transformation of ovarian endometrioma. While she hesitated and turned to receive two cycles of oocyte retrieval because of nulliparity. Meanwhile, more papillary growth in the ovarian endometrioma with intratumor flow was found during follow-up. Laparoscopic enucleation was performed later, and pathology revealed clear cell carcinoma with peritoneal involvement, at least FIGO stage IIB. She then underwent debulking surgery to grossly no residual tumor and received adjuvant chemotherapy with no tumor recurrence in post-operative 17-months follow-up. Considering fertility preservation, conservative treatment of ovarian endometrioma is typically indicated for those women who have not yet completed childbearing. However, malignant transformation may still occur despite long-term progestin treatment. Therefore, careful image follow-up is still indispensable.

## 1 Introduction

Endometriosis is a common disease globally with a 5 to 10% prevalence in women of reproductive age (1). Approximately 15% of Asian women are diagnosed with endometriosis, significantly more frequently than the 2 to 10% of Western populations (2–5). While considered a benign disease, those deeply infiltrative cases of endometriosis can invade nearby organs, like the gastrointestinal and genitourinary systems (1). Endometriosis also had been reported in the lung parenchyma through blood stream (6). In most cases, the conditions can be well controlled by medication or surgery (5). Women with endometriosis usually have already diminished ovarian reserve, and the surgical intervention may further diminish this reserve, despite efforts to preserve ovarian tissue (7). Therefore, for young women who wish to preserve fertility but are not planning immediate conception, conservative medical management is still the preferred initial treatment (8).

Endometriosis is also considered the precursor to epithelial ovarian malignancy, with the histological types of either clear cell carcinoma and endometrioid carcinoma (9). Oral contraceptives are proved to decrease risk of ovarian cancer mainly from the effect of progestin (10). Dienogest is an effective medical treatment as a novel progestin for endometriosis with less side effect (11). Cases of malignancy transformation of endometriosis have been reported under the medication of dienogest. However, in those case reports, malignancy typically occurs within a relatively short time after start of dienogest treatment (12). Therefore, whether or not there were occult malignancy at the initiation of diengoest was suspicious. Here, we present a case in a young lady with malignant changes of ovarian endometrioma during a prolonged use of dienogest for 5 years. We also reviewed the related literature.

## 2 Case presentation

**Figure 1:**
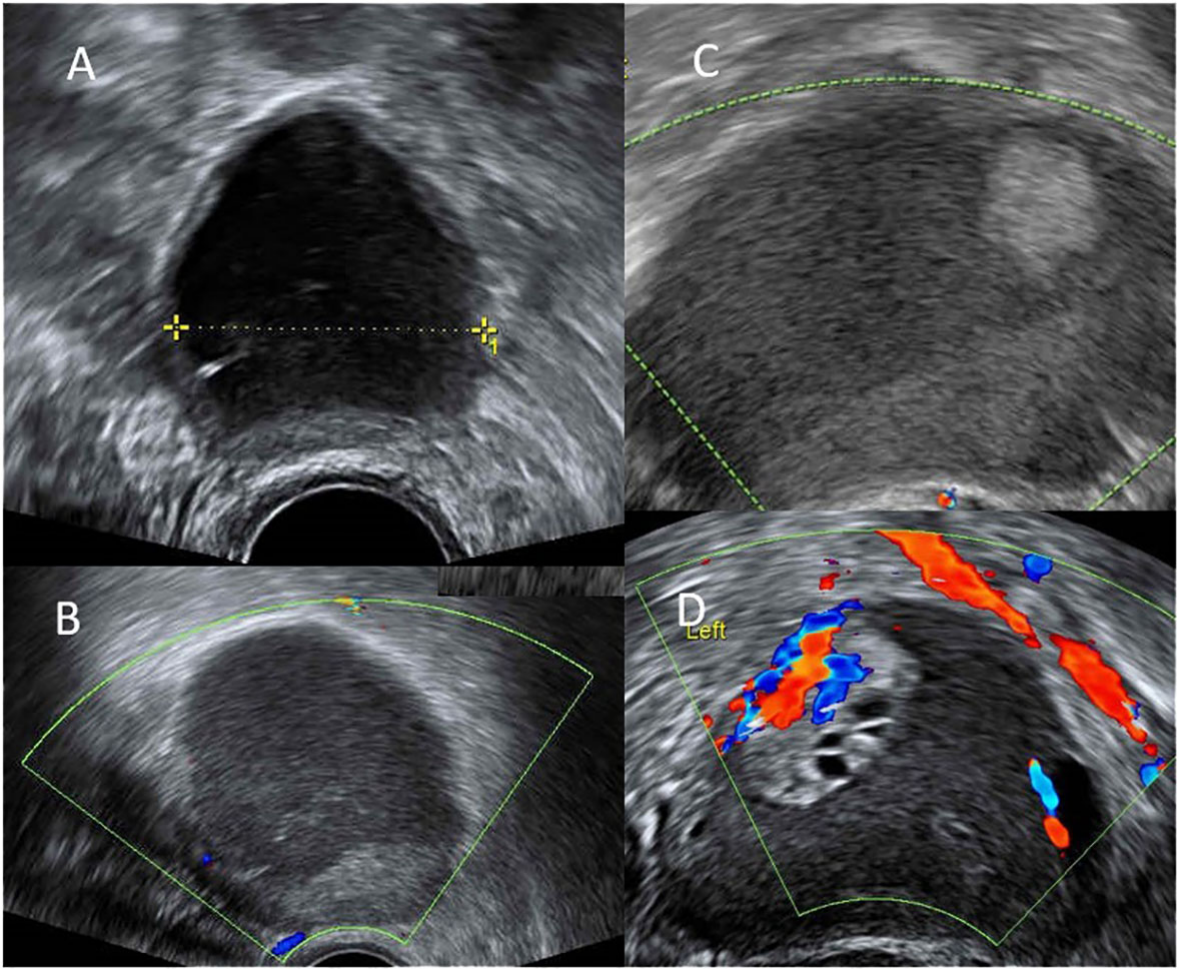
**(A)** endometrioma 6cm at the 0-month of dienogest use. **(B)** decreased size of endometrioma to 3cm with thin wall at the 24-months of dienogest use. **(C)** increased size of endometrioma to 6cm with mural part suspect malignancy at the 48-months of dienogest use. **(D)** endometrioma had increased to 8cm with excrescences 3cm and intra-tumor flow at the 51-months of dienogest use.

A 35-year-old nulliparous Taiwanese woman, without systemic underlying disease, initially developed a small asymptomatic endometrioma of 2 to 3 cm in size. Before that time, she received no medication. She suffered from acute abdominal pain when the endometrioma had increased to 6 cm (**Figure 1A**). She had been taking dienogest since the age of 30. The size of endometrioma dropped to 3 cm (**Figure 1B**) at the 24-months of dienogest use. Sonography follow-up continued every 3 months, and her endometrioma remained in a stable size.

However, at the 48-months of dienogest use, her endometrioma enlarged to 5 cm with a 1.3 cm mural nodule without internal flow (**Figure 1C**). Levels of tumor markers, including CA125 and CA199, were both within normal limits. Her anti-mullerian hormone (AMH) serum level was 0.54 ng/mL. Under the suspicion of ovarian malignancy, surgical intervention was suggested. The patient hesitated and turned to receive two cycles of oocyte cryopreservation prior to surgery. Three months later (at the 51-months of dienogest use), her endometrioma further enlarged to 8 cm, and with the presence of 3 cm excrescence showing intra-tumor flow (**Figure 1D**). The patient was still hesitant to receive surgery. Finally, at the 54-month use of dienogest, she underwent laparoscopic enucleation of the endometrioma in another medical center. Pathological report confirmed a clear cell carcinoma with peritoneal involvement, FIGO stage IIB at least. The results of immunohistochemical (IHC) staining of the tumor were Napsin A(+), HNF-1β(+), WT1 (–), ER (–), which suggested the diagnosis of clear cell carcinoma.

She came to our hospital for a second opinion. Abdominal computed tomography reported no obvious intra-abdominal metastasis, nor lymphadenopathy. After counseling, fertility preserving surgery was considered not feasible, and definitive cancer treatment was conducted with debulking surgery including hysterectomy, bilateral salpingo-oophorectomy, omentectomy, bilateral pelvic and para-aortic lymph nodes dissection with excision of trocar sites. There was no grossly residual tumor after surgery (R0 response). No residual tumor was found in her final pathological examination. She underwent two cycles of post-operative chemotherapy with paclitaxel and carboplatin. However, with a severe allergy to paclitaxel in the third cycle, the regimens were switched to cyclophosphamide and carboplatin. She has so far completed 6 cycles of postoperative adjuvant chemotherapy, and no tumor recurrence at 17-months follow up.

## 3 Discussion

This young nulliparous Taiwanese woman with a small endometrioma that increased in size despite initial response after taking dienogest for almost 5 years. Surgical intervention was later suggested for the suspicion of malignant transformation of ovarian endometrioma, while the patient turned to receive oocyte cryopreservation before surgery. She underwent laparoscopic enucleation of the endometrioma first, and clear cell carcinoma with peritoneal involvement was confirmed. Debulking surgery and post-operative chemotherapy were completed with no recurrence at 17-months follow up. Although no cytological or pathological diagnosis was made to confirm the benign nature of the endometrioma in this patient initially. However, considering her regular ultrasound assessments and the doubling time of ovarian cancer 2.5-4 months (13), and malignancy was finally diagnosed after five years of continuous dienogest use, suggesting the benign nature at the start of the treatment.

Management with medication or surgical intervention of endometriosis is often based on symptoms, fertility desire, and disease severity after comprehensive discussion with patients (5). Medication including symptoms relief agents or hormonal treatment with oral contraceptive pill, progestin, aromatase inhibitor and gonadotropin-releasing hormone agonist (GnRHa) (5). Surgical intervention is effective in reducing endometriosis-associated pain especially in the case of deep infiltrating endometriosis (4). But the surgery inevitably reduces ovarian reserve despite careful preservation of ovarian tissue (7). Moreover, women with endometriosis usually have diminished ovarian reserve (7), thus medication control is often the choice for treating young ladies who have not completed childbearing. Dienogest is a 4th-generation selective progestin for oral use. It is a derivative of 19-nortestosterone, that has been widely used for endometriosis treatment (14) and is able to reduce the size of endometrioma (11). The efficacy and tolerability of dienogest are similar to the gonadotropin-releasing hormone agonist (GnRHa), with fewer adverse side effects including menopausal-like symptoms and reduced bone mineral density during prolonged use (15).

From the Pubmed search on long-term use of dienogest, we found no report on malignant transformation of endometriosis. Only a case series of malignant transformation of endometrioma within a relatively shorter duration of dienogest was reported in Japan (12). The 4 cases with dienogest treatment were within ages from 42 to 44 years, all nullipara, and malignancy were found at 42 to 46 years old. Their durations of taking dienogest covered 9 to 33 months. Their cyst size before dienogest was between 3 and 8 cm, and the cyst size at malignant transformation was between 2 and 7 cm. Pathology types were all clear cell carcinoma, staging 1C1-1C3 (**Table 1**). They presumed characteristics for high risks of malignant transformation include advanced age, nulliparity, and recurrence with significantly growing cyst size (12). In our case, despite her younger age and longer duration of the dienogest control, and no significant change in size comparing to initial diagnosis, her malignancy still occurred at a more advanced stage.

**Table 1:** Case summary of malignancy change of endometrioma under dienogest in review of literature.

	Case 1 (2019 Honda et al.) (12)	Case 2 (2019 Honda et al.) (12)	Case 3 (2019 Honda et al.) (12)	Case 4 (2019 Honda et al.) (12)	Case 5 (this case)
Parity	Nullipara	Nullipara	Nullipara	Nullipara	Nullipara
Age at start of dienogest	43 years	44 years	42 years	44 years	30 years
Age at malignant transformation	44 years	46 years	42 years	46 years	35 years
Cyst size before dienogest	7 cm	8 cm	4 cm	3 cm	6 cm
Cyst size at malignant transformation	7 cm	5 cm	7 cm	3 cm	5cm
Duration of dienogest	14 months	31 months	9 months	33 months	54months
Operation for ovarian carcinoma	TAH, BSO, OM, PLA, PALA	LSO	TAH, BSO, OM, PLA, PALA	TAH, BSO, OM	TAH, BSO, OM, PLA, PALA
Pathology	Clear cell carcinoma	Clear cell carcinoma	Clear cell carcinoma	Clear cell carcinoma	Clear cell carcinoma
FIGO stage	1C1	1C3	1C1	1C1	IIB
Chemotherapy	TC for 6 cycles	TC for 3 cycles	TC for 6 cycles	TC for 6 cycles	TC for 2 cycles, shifted to cyclophosphamide and carboplatin for 4 more cycles
Serum CA 125 at malignant transformation	515 U/ml	78.5 U/ml	42.4 U/ml	46.3 U/ml	30.26 U/ml

BSO, Bilateral salpingo-oophorectomy; CA, 125 Cancer antigen 125; FIGO, International Federation of Gynecology and Obstetrics; LSO, Left salpingo-oophorectomy; OM, Omentectomy; PALA, Para-aortic lymphadenectomy; PLA, Pelvic lymphadenectomy; TAH, Total abdominal hysterectomy; TC, Paclitaxel and carboplatin.

The lifetime risk of ovarian cancer in women is 1.31 to 1.4% in the general population (16, 17). Patients with ovarian endometrioma have a slightly higher risk (1.8%) of ovarian cancer (17). Endometriosis-associated ovarian cancers are most commonly with the histopathological type of clear cell carcinoma and endometrioid carcinoma (18), with significant genetic correlation was reported at previous meta-analysis (19). Many studies have demonstrated that women with endometriosis have an increased risk of ovarian malignancy (18, 20). A cohort study in Taiwan demonstrated that Taiwanese women with endometriosis had three-fold increase of risk in newly developed epithelial ovarian cancer (21). Endometriosis-associated ovarian cancers are believed to develop from ovarian atypical endometriosis component (22). Kosuke Murakami et al. suggests that those clinically detectable cysts subsequently diagnosed as ovarian cancer likely contain pre-existing cancer cells (23).

Model of epithelial ovarian carcinogenesis classified endometriosis-related tumors, including endometrioid and clear cell carcinomas, as type I tumors, which develop from benign extraovarian lesions that implant in the ovary and subsequently undergo malignant transformation (24). There is substantial evidence supporting the association between endometriosis and clear cell carcinoma. Endometriosis triggers inflammation and hormone production, fostering an environment conducive to ovarian cancer. Genetic alterations accumulate over time, leading to endometriosis-associated malignant transformation. This progression starts with abnormal epithelial growth, develops into borderline tumors, and ultimately results in ovarian cancer (25). Histopathologic examination usually revealed coexistence of benign endometrioma with atypical endometriosis and ovarian malignancy (26, 27). Furthermore, endometriosis is found in over 50% of ovarian clear cell carcinoma (28). Recent research has identified several key molecular drivers in the malignant transformation from endometriosis to clear cell carcinoma, including ARID1A mutations, PIK3CA mutations, inactivating PTEN mutations and HNF-1β activation (24, 29). The ongoing advancements in genomics and molecular biology are poised to uncover additional mechanisms underlying the malignant transformation of endometriosis (30).

Oral contraceptive progestin appears to create protection from ovarian cancer (10), and to induce apoptosis in ovarian epithelium in animal, which could prevent the development of ovarian cancer in view of the apoptosis pathway for cancer prevention (31). A meta-analysis demonstrated a significant duration-response relationship with reduction in incidence of more than 50% among women using oral contraceptives for 10 or more years in primary prevention (10). Dienogest, as a progestin, can in theory prevent the malignant transformation of endometriosis (32). Nakamura et al. reported that dienogest is an orally active antagonist of angiogenesis, and its antiangiogenic action has effects on cancer xenografts and endometriosis *in vitro* on rats and mice (33). Dienogest also exerts local effects on endometriotic lesions and inhibits cytokine secretions of the stroma of endometrial cells, resulting in antiproliferative effects (34).

## 4 Conclusions

The management of endometriosis in young women of reproductive age is a challenge for gynecologists. As increasingly number of women postpones their childbirth, at the same time decrease of ovarian reserve due to aging, conservative treatment without excision of ovarian endometrioma is typically the choice of treatment. A balance between disease control and fertility preservation should be considered. Malignant transformation may still may occur during long-term maintenance treatment. Careful follow-up and timing intervention when malignancy was suspected should still be emphasized.

## Data availability statement

The original contributions presented in the study are included in the article/supplementary material. Further inquiries can be directed to the corresponding author.

## Ethics statement

The studies involving humans were approved by the Institutional Review Board-CE23325A of Taichung Veterans General Hospital, Taichung, Taiwan. The studies were conducted in accordance with the local legislation and institutional requirements. The participants provided their written informed consent to participate in this study. Written informed consent was obtained from the individual(s) for the publication of any potentially identifiable images or data included in this article.

## Author contributions

YC: Writing – original draft, Writing – review & editing. TL: Writing – review & editing. LS: Writing – review & editing. YS: Writing – review & editing. SH: Writing – review & editing. CL: Writing – review & editing. SH: Writing – review & editing. CL: Conceptualization, Supervision, Writing – review & editing.
